# Nationwide Increase in Complex Congenital Heart Diseases After the Fukushima Nuclear Accident

**DOI:** 10.1161/JAHA.118.009486

**Published:** 2019-03-13

**Authors:** Kaori Murase, Joe Murase, Akira Mishima

**Affiliations:** ^1^ Nagoya City University Graduate School of Natural Sciences Nagoya Japan; ^2^ Goshawk Protection Fund Utsunomiya Japan; ^3^ Department of Cardiovascular Surgery Nagoya City University Graduate School of Medical Sciences Nagoya Japan

**Keywords:** congenital heart disease, Fukushima nuclear accident, neonates and infants, Congenital Heart Disease, Epidemiology, Pediatrics

## Abstract

**Background:**

After the Chernobyl nuclear accident in 1986, an increase in the incidence of congenital heart disease (CHDs) in the neighboring countries was reported. In 2011, Japan experienced the Great East Japan Earthquake and the nuclear accidents at Fukushima. However, a nationwide study of their effects has not been conducted yet.

**Methods and Results:**

We used data covering the period between 2007 and 2014 from the annual surveys conducted by the Japanese Association for Thoracic Surgery, which included almost all of the operations pertaining to 46 types of CHDs in Japan. CHDs were divided into 2 groups based on complexity, the time of occurrence during heart development, and age at operation. We estimated the change in the number of the operations per 100 000 live births between pre‐ and postdisaster using a negative binomial generalized linear mixed model. Overall, a significant 14.2% (95% CI, 9.3–19.4) increase in the number of operations for complex CHDs in neonates and infants per 100 000 live births was found, whereas those performed for patients of 1 to 17 years old showed no significant change during the study period.

**Conclusions:**

The number of operations for complex CHDs in neonates and infants in Japan significantly increased after the massive disaster, and its level was maintained thereafter. The number of operations for complex CHD was not equal but closely correlated to the live birth prevalence of complex CHDs. Therefore, some meaningful increase in the live birth prevalence can be assumed; however, the precise cause of the increase is unknown.

**Clinical Perspective**

**What Is New?**

- This study investigated the influence of a huge earthquake followed by a nuclear accident on complex congenital heart diseases in terms of the number of the operations in 1‐year‐old patients.
- The nationwide impact was assessed by comparing the pre‐ and postaccident periods in Japan under the conditions of the existing well‐established medical care system and medical statistics.

**What Are the Clinical Implications?**

- A disaster associated with a nuclear accident might increase the risk of a wide range of complex congenital heart diseases.
- Establishment of a medical care system and medical statistics is required for assessing the influence of unexpected disasters.

## Introduction

On March 11, 2011, the Great East Japan Earthquake occurred followed by a great tsunami and the nuclear accidents at the Fukushima Daiichi Nuclear Power Plant. It is known that various health problems are associated with massive disasters. Reproductive outcomes, such as stillbirth, preterm birth, low birth weight, and congenital anomalies, are also affected by such disasters.^1^

The Great East Japan Earthquake was accompanied by the nuclear accidents. After the Chernobyl accident, which was also a nuclear disaster, congenital anomalies apparently increased in the countries near the disaster site, but their direct relevance to radiation exposure was considered to be minor.^2^ A paper describing the conclusion by EUROCAT (European Registry of Congenital Anomalies and Twins) showed that no detectable increase in the incidence of congenital anomalies due to ionizing radiation was observed in Western Europe, relatively far from the incident site.^3^ In terms of congenital heart anomalies, which are one of the most common congenital anomalies,^4^ a paper reported an increase in congenital heart diseases (CHDs) after the Chernobyl accident.^5^ However, the study did not receive a positive reception because of the lack of methodological details.^6^, ^7^

What was the situation in Japan after the massive disaster in 2011? The Fukushima Health Management Survey launched by the Fukushima prefecture reported no changes in reproductive outcome compared with the recent averages elsewhere in Japan.^8^ However, the nationwide trend, including the adjacent prefectures, was not studied. The survey may have underestimated the impact of the disaster because the evacuees’ were not fully investigated. Furthermore, patient discharge rates after surgery for cryptorchidism, which is a congenital abnormality, had increased nationwide (13.4%; 95% CI, 4.7–23.0%) after the Fukushima nuclear accidents in 2011.^9^ As a result, we wondered whether there was a similar tendency for CHDs. We focused our attention on the data collected by the Japanese Association for Thoracic Surgery. The data can be considered as an entire patient population enabling comparison of the pre‐ and postdisaster periods because the association has been recording almost all operations for CHD in Japan since before the disaster in 2011.^10^ Because Japan provides universal healthcare coverage,^11^ the number of surgeries in Japan is independent of the patients’ socioeconomic level. This would help minimize the bias in the data. Another advantageous point is that in contrast to the Chernobyl study, the number of specific CHD operations was recorded. This enables us to examine the association between the disaster and the type of CHD. When defects occur in the early stages of heart development, the CHDs tend to be complex and severe, and require difficult surgical treatment; consequently, the incidence trends of such complex CHDs are an area of concern. Thus, in this paper, we examined the change in the number of operations performed for CHDs, dividing the CHDs into complex and noncomplex.

## Methods

The data and analytic methods that support the findings of this study are available from the corresponding author upon reasonable request.

### Study Population and Data Sources

We used the data from the annual surveys conducted by the Japanese Association for Thoracic Surgery since 1986.^10^ The surveys cover almost all of the surgeries pertaining to CHD in Japan. The association sends out questionnaires to almost all institutions, receives the answers and compiles the annual report. In this study, we considered the 4 years before and after the Great East Japan Earthquake on March 11, 2011 (ie, 2007–2014). Response rates during the period were 95.2% (558 of 586; 2007), 99.0% (593 of 599; 2008), 97.5% (586 of 601; 2009), 99.0% (586 of 592; 2010), 96.4% (570 of 591; 2011), 97.0% (583 of 601; 2012), 97.8% (589 of 602; 2013), and 97.1% (561 of 578; 2014). The figures in parentheses represent the number of the institutes that received and answered the questionnaire each year.

These data contained the number of operations pertaining to CHD, whether cardiopulmonary bypass (CPB) was used, age (neonate, infant, 1–17 years, ≥18 years), and surgical classification (50 types, including 4 types of “redo”; these types were characterized according to the complexity of the operative procedure and the need for additional operations). We analyzed 46 surgical classifications, excluding the redo operations and those in patients under 1 year old (neonate and infant), regardless of the use of CPB, in order to infer the change in the incidence (more accurately, live birth prevalence) of CHD. The numbers were aggregated according to the surgical classification and year.

### Congenital Heart Diseases

Different types of cardiovascular malformation occur according to the developmental stage of their responsible anomaly.^12^ Generally, the morphological aberration tends to be more serious when occurring at an earlier developmental stage. In this study, we classified the surgical classifications according to pre‐ and postorganogenesis period, except for “coronary disease” and “others.” The surgical classifications were also classified according to the complexity of the malformation (complex or noncomplex malformation) except for “aneurysms of the sinus of Valsalva,” “aortic valve lesion,” “mitral valve lesion,” “coronary disease,” and “others.” The classification is shown in Table 1.

**Table 1:** Categorization of Congenital Heart Diseases According to Developmental Stage and Complexity

No.	Surgical Classification^a^	Onset	Type	No.	Surgical Classification	Onset	Type
1	PDA	L	S	26	VSD (subarterial)	L	S
2	Coarctation (simple)	E	S	27	VSD (perimemb/muscular)	L	S
3	Coarctation+VSD	E	C	28	VSD+PS	E	S
4	Coarctation+DORV	E	C	29	DCRV±VSD	E	C
5	Coarctation+AVSD	E	C	30	Aneurysm of sinus of Valsalva	L	O
6	Coarctation+TGA	E	C	31	TOF	E	C
7	Coarctation+SV	E	C	32	PA+VSD	E	C
8	Coarctation+others	E	O	33	DORV	E	C
9	Interrupt. of Ao (simple)	E	C	34	TGA (simple)	E	C
10	Interrupt. of Ao+VSD	E	C	35	TGA+VSD	E	C
11	Interrupt. of Ao+DORV	E	C	36	TGA VSD+PS	E	C
12	Interrupt. of Ao+Truncus	E	C	37	Corrected TGA	E	C
13	Interrupt. of Ao+TGA	E	C	38	Truncus arteriosus	E	C
14	Interrupt. of Ao+others	E	C	39	SV	E	C
15	Vascular ring	E	S	40	TA	E	C
16	PS	E	S	41	HLHS	E	C
17	PAIVS or critical PS	E	S	42	Aortic valve lesion	E	O
18	TAPVR	E	C	43	Mitral valve lesion	E	O
19	PAPVR±ASD	E	S	44	Ebstein	E	C
20	ASD	L	S	45	Coronary disease	O	O
21	Cor triatriatum	E	C	46	Others	O	O
22	AVSD (partial)	E	S	47	Redo VSD	R	···
23	AVSD (complete)	E	C	48	Redo VSD PS release	R	···
24	AVSD+TOF or DORV	E	C	49	Redo VSD RV‐PA conduit replace	R	···
25	AVSD+others	E	C	50	Redo VSD Others	R	···

ASD indicates atrial septal defect; AVSD, atrioventricular septal defect; C, complex; DCRV, double‐chambered right ventricle; DORV, double‐outlet right ventricle; E, early; HLHS, hypoplastic left heart syndrome; Interupt. of Ao, interruption of aorta; L, late; O, other; PAIVS, pulmonary atresia with intact ventricular septum; PAPVR, partial anomalous pulmonary venous return; PDA, patient ductus arteriosus; PS, pulmonary stenosis; R, redo; RV‐PA, right ventricle‐pulmonary artery; S, simple; SV, single ventricle; TA, tricuspid atresia; TAPVR, total anomalous pulmonary venous return; TGA, transposition of great arteries; TOF, tetralogy of Fallot; VSD, ventricular septal defect.

a The names of the surgical classification and numbers are based on the annual reports published by the Japanese Association for Thoracic Surgery.

### Yearly Change in the Number of Operations

To analyze the yearly change in the total number of CHD operations, we calculated the total number of operations per 100 000 live births each year. The 95% CIs were also calculated assuming the numbers followed a Poisson distribution. Moreover, we separated the 46 CHDs into “early” and “nonearly” categories according to their developmental stage, and the early CHDs were also divided into “complex” and “noncomplex” as described in Table 1. We also calculated the number of the operations per 100 000 live births and 95% CIs for each division in the same way. The number of live births was excerpted from the Population Survey Report published by the Ministry of Health, Labour, and Welfare.^13^ Additionally, to review the yearly changes in the 46 CHDs, we calculated the number of operations per 100 000 live births for each surgical classification and accumulated them in ascending order by the 8‐year total numbers of respective surgical classifications. We also calculated the number of complex and nonearly operations in patients 1 to 17 years old and examined the change and deviation in trend in order to determine the relation between the number of the operations for ages 0 to 1 years and that for 1 to 17 years.

### Statistical Analysis

We performed a generalized linear mixed model (GLMM) analysis to examine the change in the number of operations between the predisaster period (2007–2010) and postdisaster period (2011–2014). The analysis employed a “log” link function and contained a random intercept and a random change rate in the numbers between the pre‐ and postdisaster period for each CHD. We performed Poisson GLMM and a negative‐binomial GLMM analysis to determine the appropriate distribution for the data. We employed the “aods3” package of R to assess the goodness of fit of the models. Subsequently, we also performed a Bayesian analysis using the appropriate distribution as a sensitivity analysis (Data S1). Moreover, we performed a Cochran‐Armitage trend test to examine whether a trend existed since the predisaster period and examined the deviation if such a significant trend were detected. When a significant deviation was detected, multiple comparisons according to the methods of Benjamini and Yekutieli^14^ were also performed. We set the level of significance at 0.05 for the trend test and subsequent tests.

We performed additional GLMM analyses according to the time of development.^15^, ^16^ The data we used in this study were from the Society of Thoracic Surgery in Japan, and they were classified according to the type of CHD surgery. Some types of CHDs classified according to surgery type fit the descriptions of multiple types of CHDs classified according to the time of development. The corresponding relationships are shown in Tables 2 and 3. Interruption of the aortic arc was classified as type A or type B according to the location of interruption (type A and type B correspond to left ventricular outflow tract obstruction and conotruncal, respectively); however, this classification was not used for our data. We divided the number of interruption of aortic arc surgeries into 70% for left ventricular outflow tract obstruction and 30% for conotruncal because the interruption of aortic arc type ratio is 7:3 (type A:type B) in Japan.

**Table 2:** Correspondence Between Surgical Classification in Japan and Developmental Classification of CHDs According to Yamagishi (2007)^15^

No.	Surgical Classification	Developmental Classification		
1	PDA	Great artery		
2	Coarctation (simple)	Great artery		
3	Coarctation+VSD	Ventricular septum	Conotruncal	Great artery
4	Coarctation+DORV	Cardiac looping	Conotruncal	Great artery
5	Coarctation+AVSD	Endocardial cushion	Great artery	
6	Coarctation+TGA	Conotruncal	Great artery	
7	Coarctation+SV	Ventricle	Great artery	
8	Coarctation+others	Great artery		
9	Interrupt. of Ao (simple)	Great artery		
10	Interrupt. of Ao+VSD	Ventricular septum	Conotruncal	Great artery
11	Interrupt. of Ao+DORV	Cardiac looping	Conotruncal	Great artery
12	Interrupt. of Ao+Truncus	Conotruncal	Great artery	
13	Interrupt. of Ao+TGA	Conotruncal	Great artery	
14	Interrupt. of Ao+others	Great artery		
15	Vascular ring	Great artery		
16	PS	Aortic/pulmonary valve		
17	PAIVS or critical PS	Aortic/pulmonary valve		
18	TAPVR	Atrium/atrial septum		
19	PAPVR±ASD	Atrium/atrial septum		
20	ASD	Atrium/atrial septum		
21	Cor triatriatum	Atrium/atrial septum		
22	AVSD (partial)	Endocardial cushion		
23	AVSD (complete)	Endocardial cushion		
24	AVSD+TOF or DORV	Cardiac looping	Endocardial cushion	Conotruncal
25	AVSD+others	Endocardial cushion		
26	VSD (subarterial)	Ventricular septum	Conotruncal	
27	VSD (perimemb/muscular)	Ventricular septum	Conotruncal	
28	VSD+PS	Ventricular septum	Conotruncal	Aortic/pulmonary valve
29	DCRV±VSD	Ventricular septum	Conotruncal	
30	Aneurysm of sinus valsalva	Coronary artery		
31	TOF	Conotruncal		
32	PA+VSD	Ventricular septum	Conotruncal	Aortic/pulmonary valve
33	DORV	Cardiac looping	Conotruncal	
34	TGA (simple)	Conotruncal		
35	TGA+VSD	Ventricular septum	Conotruncal	
36	TGA VSD+PS	Ventricular septum	Conotruncal	Aortic/pulmonary valve
37	Corrected TGA	Cardiac looping		
38	Truncus arteriosus	Conotruncal		
39	SV	Ventricle		
40	TA	Cardiac looping		
41	HLHS	Ventricle		
42	Aortic valve lesion	Aortic/pulmonary valve		
43	Mitral valve lesion	Endocardial cushion		
44	Ebstein	Endocardial cushion		
45	Coronary disease	Coronary artery		
46	Others	Others		
47	Redo VSD	Redo		
48	Redo VSD PS release	Redo		
49	Redo VSD RV‐PA conduit replace	Redo		
50	Redo VSD others	Redo		

ASD indicates atrial septal defect; AVSD, atrioventricular septal defect; DCRV, double‐chambered right ventricle; DORV, double‐outlet right ventricle; HLHS, hypoplastic left heart syndrome; Interupt. of Ao, interruption of aorta; PAIVS, pulmonary atresia with intact ventricular septum; PAPVR, partial anomalous pulmonary venous return; PDA, patient ductus arteriosus; PS, pulmonary stenosis; RV‐PA, right ventricle‐pulmonary artery; SV, single ventricle; TA, tricuspid atresia; TAPVR, total anomalous pulmonary venous return; TGA, transposition of great arteries; TOF, tetralogy of Fallot; VSD, ventricular septal defect.

**Table 3:** Correspondence Between Surgical Classification in Japan and Developmental Classification of CHDs According to Botto et al (2007)^16^

No.	Surgical Classification	Developmental Classification		
1	PDA	Excluded		
2	Coarctation (simple)	LVOTO		
3	Coarctation+VSD	LVOTO	Septal	
4	Coarctation+DORV	Conotruncal		
5	Coarctation+AVSD	AVSD		
6	Coarctation+TGA	Conotruncal		
7	Coarctation+SV	LVOTO	Complex	
8	Coarctation+others	LVOTO		
9	Interrupt. of Ao (simple)	LVOTO	Conotruncal	
10	Interrupt. of Ao+VSD	LVOTO	Conotruncal	Septal
11	Interrupt. of Ao+DORV	LVOTO	Conotruncal	
12	Interrupt. of Ao+Truncus	LVOTO	Conotruncal	
13	Interrupt. of Ao+TGA	LVOTO	Conotruncal	
14	Interrupt. of Ao+others	LVOTO	Conotruncal	
15	Vascular ring	Excluded		
16	PS	RVOTO		
17	PAIVS or critical PS	RVOTO		
18	TAPVR	APVR		
19	PAPVR±ASD	APVR		
20	ASD	Septal		
21	Cor triatriatum	Excluded		
22	AVSD (partial)	AVSD		
23	AVSD (complete)	AVSD		
24	AVSD+TOF or DORV	AVSD		
25	AVSD+others	AVSD		
26	VSD (subarterial)	Septal		
27	VSD (perimemb/muscular)	Septal		
28	VSD+PS	RVOTO	Septal	
29	DCRV±VSD	Excluded		
30	Aneurysm of sinus of Valsalva	Excluded		
31	TOF	Conotruncal		
32	PA+VSD	RVOTO		
33	DORV	Conotruncal		
34	TGA (simple)	Conotruncal		
35	TGA+VSD	Conotruncal		
36	TGA VSD+PS	Conotruncal		
37	Corrected TGA	Complex		
38	Truncus arteriosus	Conotruncal		
39	SV	Complex		
40	TA	RVOTO		
41	HLHS	LVOTO		
42	Aortic valve lesion	Excluded		
43	Mitral valve lesion	Excluded		
44	Ebstein	RVOTO		
45	Coronary disease	Excluded		
46	Others	Excluded		
47	Redo VSD	Excluded		
48	Redo VSD PS release	Excluded		
49	Redo VSD RV‐PA conduit replace	Excluded		
50	Redo VSD others	Excluded		

APVR indicates anomalous pulmonary venous return; ASD, atrial septal defect; AVSD, atrioventricular septal defect; DCRV, double‐chambered right ventricle; DORV, double‐outlet right ventricle; HLHS, hypoplastic left heart syndrome; Interupt. of Ao, interruption of aorta; LVOTO, left ventricular outflow tract obstruction; PAIVS, pulmonary atresia with intact ventricular septum; PAPVR, partial anomalous pulmonary venous return; PDA, patient ductus arteriosus; PS, pulmonary stenosis; RVOTO, right ventricular outflow tract obstruction; RV‐PA, right ventricle‐pulmonary artery; SV, single ventricle; TA, tricuspid atresia; TAPVR, total anomalous pulmonary venous return; TGA, transposition of great arteries; TOF, tetralogy of Fallot; VSD, ventricular septal defect.

We obtained study approval from the Institutional Review Board of Nagoya City University. The board waived the requirement for informed consent due to the anonymous nature of the data.

## Results

**Figure 1:**
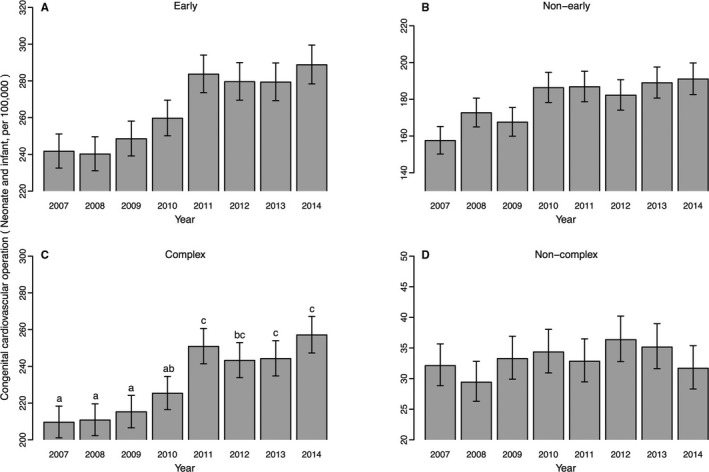
Number of operations for congenital heart disease in Japan. The yearly change in the operations for congenital heart disease per 100 000 births (under the age of 1, excluding “redo” classifications) in Japan is shown. The error bars indicate 95% CIs assuming the total numbers follow the Poisson distribution. **A**, The 39 “early” developmental stage diseases. **B**, The 7 “nonearly” developmental stage diseases. **C**, The 29 “complex” diseases. **D**, The 10 “noncomplex” diseases. The result of multiple comparisons is indicated by the letters in (**C**).

The yearly change in the number of operations for CHDs per 100 000 live births is shown in Figure 1. The data used are shown in Table S1. The early group showed a remarkable increase in 2011 (Figure 1A), while the nonearly group showed a monotonic increase from the predisaster period with no significant deviation (Figure 1B, Cochran‐Armitage trend test: *P*<0.001; deviations from the linear trend: *P*=0.161). The majority of the early CHDs were complex, and the yearly change in early CHDs was largely accounted for by the complex CHDs (Figure 1C), whereas noncomplex CHDs seemed to have no specific tendency (Figure 1D). The complex CHDs showed a nonmonotonic increase (Figure 1C; Cochran‐Armitage trend test: *P*<0.001; deviations from the linear trend: *P*<0.05). Combined with the result of the multiple comparisons, a large increase between 2010 and 2011 was demonstrated and the increased level was maintained.

**Figure 2:**
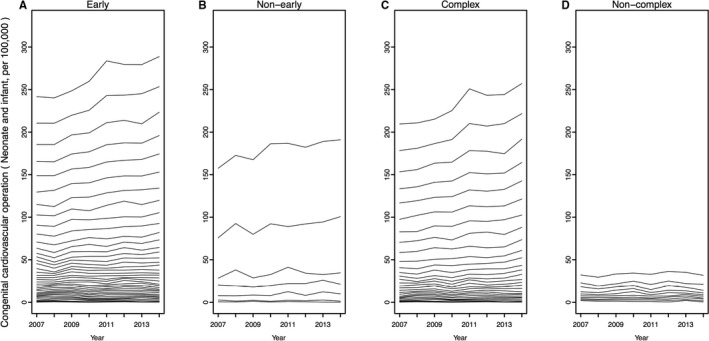
Number of operations for congenital heart disease (cumulative). The cumulative yearly change in operations for congenital heart disease per 100 000 births (under the age of 1, excluding “redo” classifications) is shown. The numbers are accumulated in ascending order by the 8‐year total number of each classification. **A**, The 39 “early” developmental stage diseases. **B**, The 7 “nonearly” developmental stage diseases. **C**, The 29 “complex” diseases. **D**, The 10 “noncomplex” diseases.

The large increase consisted of small increases in several CHDs rather than a large increase in a few particular CHDs (Figure 2C) whereas in the case of the nonearly CHDs, a few major CHDs, ventricular septal defect (VSD; perimembranous/muscular) and patent ductus arteriosus, comprised a majority of the increase (Figure 2B).

**Figure 3:**
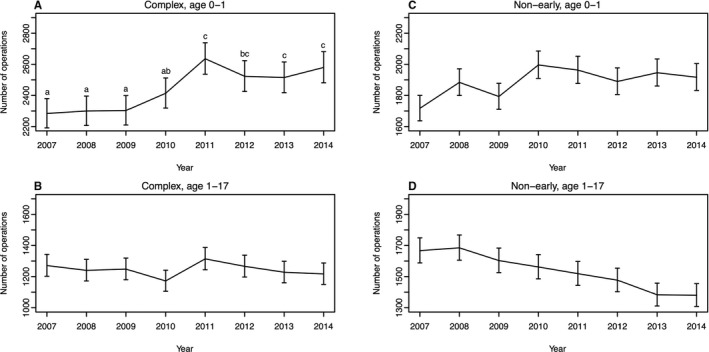
Comparison of the number of operations for those of ages 0 to 1 and 1 to 17 years. Yearly changes in the number of operations for complex or nonearly CHDs in patients 0 to 1 or 1 to 17 years are shown. **A**, Complex CHDs in those 0 to 1 years. **B**, Complex CHDs in those 1 to 17 years. **C**, Nonearly CHDs in those 0 to 1 years. **D**, Nonearly CHDs in those 1 to 17 years. The result of multiple comparison is indicated by the letters in (**A**), in which significant deviations from a linear trend were detected. CHD indicates congenital heart disease.

When we compared the number of operations for complex CHDs between the ages of 0 and 1 (Figures 1C and 3A) and 1 to 17 years (Figure 3B), the former showed a maintained rate of change after 2011 while the latter showed no changes (Figure 3B; Cochran‐Armitage trend test: *P*=0.456; deviations from the linear trend: *P*=0.119). In contrast, in the case of nonearly CHDs, operations at the age of 0 to 1 year showed a monotonic increase (Figure 3C; Cochran‐Armitage trend test: *P*<0.001; deviations from the linear trend: *P*=0.161) and those at 1 to 17 years showed a monotonic decrease (Figure 3D; Cochran‐Armitage trend test: *P*<0.001; deviations from the linear trend: *P*=0.555).

**Figure 4:**
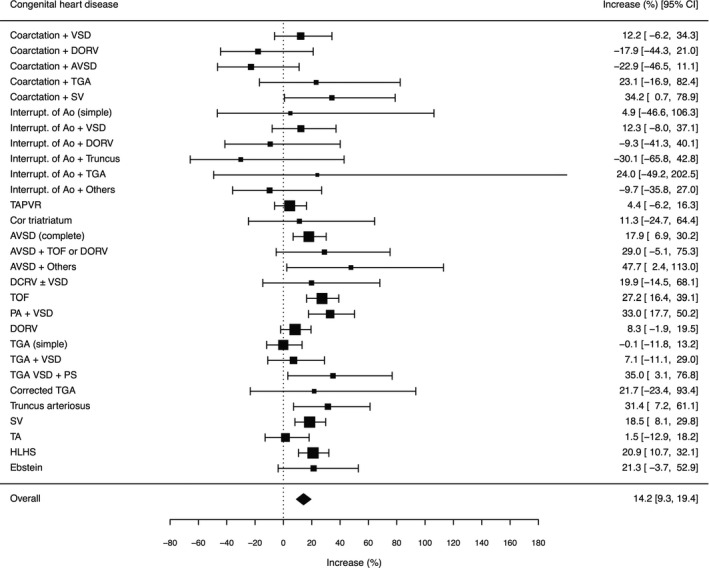
Change in the number of operations after the earthquake in 2011. The increase of the number of operations for complex CHDs between 2007–2010 and 2011–2014 is shown. The result for each classification was estimated using negative binomial GLM and the overall increase was estimated using negative binomial GLMM. AVSD indicates atrioventricular septal defect; DCRV, double‐chambered right ventricle; DORV, double‐outlet right ventricle; GLM, generalized linear model; GLMM, generalized linear mixed model; HLHS, hypoplastic left heart syndrome; Interupt. of Ao, interruption of aorta; PS, pulmonary stenosis; SV, single ventricle; TA, tricuspid atresia; TAPVR, total anomalous pulmonary venous return; TGA, transposition of the great arteries; TOF, tetralogy of Fallot; VSD, ventricular septal defect.

The goodness‐of‐fit test indicated that the negative binomial GLMM fit the data better (χ^2^=236.5, *df*=227, *P*=0.32) in comparison to the Poisson GLMM (χ^2^=286.7, *df*=228, *P*<0.01). Therefore, the negative binomial GLMM, was considered to be more appropriate for analyzing the data. The result of the negative binomial GLMM analysis of the change in the number of operations is shown in Figure 4. Overall, a 14.2% (95% CI, 9.3–19.4) significant increase was estimated, and 9 CHDs (coarctation+single ventricle, atrioventricular septal defect [AVSD; complete], AVSD+others, tetralogy of Fallot, pulmonary atresia+VSD, transposition of the great arteries+VSD+pulmonary stenosis, truncus arteriosus, single ventricle, and hypoplastic left heart syndrome) showed significant increases. A sensitivity analysis showed a consistent 14.0% (95% CI, 8.3%–19.6%; Figure S1) overall increase.

The results of GLMM based on the time of development classification are shown in Table 4. The ventricle, ventricular septum, and conotruncal groups showed a significant increase according to the classification by Yamagishi et al.^15^ However, the conotruncal, left ventricular outflow tract obstruction, septal, AVSD, and complex groups showed a significant increase according to the classification by Botto et al.^16^

**Table 4:** Results of Negative Binomial GLMM Based on the Time of Development

Classification System	Group	Mean (%)	95% CI (%)	*P* Value
Yamagishi (2007)^15^	Cardiac looping	5.3	−6.5 to 18.6	0.396
	Ventricle	20.4	12.6 to 28.8	<0.001***
	Endocardial cushion	9.1	−3.8 to 23.8	0.174
	Ventricular septum	12.5	4.9 to 20.7	<0.001***
	Atrium/atrial septum	8.0	−14.9 to 37.0	0.526
	Conotruncal	11.8	5.5 to 18.5	<0.001***
	Aortic/pulmonary valve	8.7	−8.0 to 28.3	0.326
	Great artery	8.7	−1.3 to 19.7	0.089
	Coronary artery	2.6	−35.6 to 61.8	0.920
Botto et al (2007)^16^	Conotruncal	10.3	1.1 to 20.4	0.028*
	LVOTO	15.7	6.8 to 25.3	<0.001***
	RVOTO	11.5	−1.7 to 26.3	0.090
	Septal	11.5	1.6 to 22.4	0.020*
	AVSD	15.3	6.0 to 25.5	0.001**
	Complex	22.5	5.3 to 42.5	0.009**
	APVR	0.3	−11.7 to 14.1	0.959

The “group” column indicates the disease group defined by Yamagishi (2007) or Botto et al (2007). APVR indicates anomalous pulmonary venous return; AVSD, atrioventricular septal defect; LVOTO, left ventricular outflow tract obstruction; RVOTO, right ventricular outflow tract obstruction.

Significances are represented by: ***P<0.001, **P<0.01, and *P<0.05.

## Discussion

The number of operations for CHD in neonates and infants per 100 000 live births in Japan, primarily for complex CHDs, increased by ≈14% in 2011, the year of the Great East Japan Earthquake, and that level was maintained over the following 3 years. Both congenital malformations, CHDs and cryptorchidism,^9^ showed significant increases after the Fukushima nuclear accident. The complex CHDs that showed significant increases were those known to occur during various developmental stages of the heart (eg, single ventricle or hypoplastic left heart syndrome: very early; tetralogy of Fallot: relatively early),^12^ and no particular tendency was found. The analyses based on the time of development showed that a broad range of disease groups was affected, which suggested that whole heart development would be impaired.

**Figure 5:**
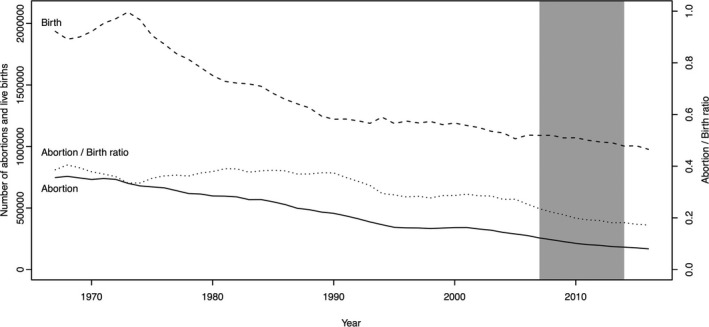
Relationship between the population abortion and live birth (50 years, from 1967 to 2016, Ministry of Health, Labour, and Welfare in Japan). Abortion indicates the number of artificial abortions (solid line). Birth indicates the number of live births in Japan (dashed line). Dotted line indicates the ratio of the number of abortions to the number of live births. The shaded area indicates the study period.

In the case of operations for complex CHD for patients 1 to 17 years old, no significant changes were detected during the study period. Thus, it would be reasonable to assume that there is no association between the number of operations performed in each age group. The ratio of the number of abortions to the number of live births during the study period in Japan decreased slowly and monotonically (Figure 5). No new guidelines were created or changed during our study period. Therefore, because there were no major changes in the screening, referral, indication for surgery, or treatment of complex CHDs during the study period in Japan, and because the institutions performing these surgeries had answered the questionnaire before 2011, the incidence of complex CHDs would have to have increased since 2011.

In contrast, in the case of the nonearly CHDs, there appeared to be an association between the monotonic increase in patients 0 to 1 year old (Figure 3C) and the monotonic decrease in those 1 to 17 years old (Figure 3D). Operation for VSD, one of the most common nonearly CHDs, is currently being performed earlier. This might explain the increase and decrease.

What caused the increase in the incidence of complex CHDs since the disaster? Given the data, we may hypothesize that the damage to gametes or the developing embryo/fetus from radionuclides emitted from the Fukushima nuclear accident was a direct factor that caused the increase. However, we have no other choice but to state that the association between radionuclides and complex CHDs is unknown at this moment because no data on individual exposure levels is available.

As an indirect effect, maternal stress (job loss, divorce, separation, or death of a close relative or friend)^17^, ^18^ is known to be one of the risk factors of conotruncal CHDs (ie, some complex CHDs). Because such stressful events were likely to have occurred during the course of the disaster, maternal stress is considered to be one of the possible causes of this increase. Another possible risk factor is diabetes mellitus. Several types of CHD have been associated with maternal diabetes mellitus,^19^ which may explain the increased incidence of single ventricle or hypoplastic left heart syndrome as well as conotruncal CHDs. Some studies have reported that diabetes mellitus has increased in the evacuees over the age of 40,^20^, ^21^ but another showed no significant change in pregnant women.^22^ Thus, it is currently unknown whether pregestational or gestational diabetes mellitus has increased in the disaster‐affected or adjacent areas. There may also be other possible factors, and further studies will be needed.

### Strengths and Limitations

Because of the universal health insurance coverage in Japan,^11^ infants with CHDs are treated properly regardless of their family's socioeconomic level. This is a unique characteristic of our data in comparison to other countries’. Moreover, almost all of the institutions performing surgeries for CHD had answered the questionnaire before 2011. Thus, the data we used in this study were almost that of the entire Japanese patient population undergoing surgeries pertaining to CHDs in Japan and are considered to have little bias. Bias attributable to the compositional change of the participating institutions would also be unlikely.

Our study does have some limitations. First, the significant increase was estimated using the number of operations, not the incidence. We should note that the increase in the number of operations is higher than that of the incidence of complex CHDs because complex CHDs often require multiple operations within the first year of life. Second, the data we used in this study lack regional information and data on the exposure level of individuals. More specific patient data such as time, location, and amount of maternal exposure are required to determine the influence of radionuclides and regional difference. If the exposure were increased in a particular region, the number of operations would be greater in the region in question. Similarly, if the date of the operation or the weeks of pregnancy at the delivery day are available, we may be able to specify the event responsible for the increase in complex CHDs. Third, we were not able to identify the starting point of the increase because the data were compiled annually. If the cause of the increase had occurred in March 2011, and the fetus was in the organogenesis period, then the infants at risk would be born at the beginning of October in full term or September in moderate or late preterm. If the increase had begun before September 2011, we would not be able to attribute the increase to the disaster alone.

## Conclusions

The number of operations for complex CHDs in neonates and infants per 100 000 live births in Japan increased by ≈14% after the Great East Japan Earthquake. The increase in the incidence of complex CHDs would be lower than that of the number of operations because complex CHDs often require multiple surgical interventions within the first year of life, but some meaningful increase in the incidence may still be assumed. The cause of the increase is unknown, but we should consider the influence of the radionuclides emitted from the Fukushima nuclear power plant. More specific patient data such as time, location, and amount of maternal exposure would be required to determine the cause.

## Sources of Funding

This work was supported by the Japan Society for the Promotion of Science (KAKENHI Grant Number 16K00575), which had no role in study design, data collection, data analysis, interpretation, or approval of the manuscript. This work was supported by Grant‐in‐Aid for Research in Nagoya City University in 2018 (grant number 7).

## Disclosures

None.

## Supporting information


**Data S1.** Supplemental methods.
**Table S1.** Number of Operations for Congenital Heart Disease in Japan
**Figure S1.** Result of the Bayesian analysis for complex CHDs.
