# Supplementary material for: Nationwide Increase in Complex Congenital Heart Diseases After the Fukushima Nuclear Accident
Source: J Am Heart Assoc. 2019 Mar 13;8(6):e009486. doi: 10.1161/JAHA.118.009486 (PMC6475040; doi:10.1161/JAHA.118.009486)
Supplement: Supplementary file 1 — Data S1. Supplemental methods. Table S1. Number of Operations for Congenital Heart Disease in Japan Figure S1. Result of the Bayesian analysis for complex CHDs. [file JAH3-8-e009486-s001.pdf]

SUPPLEMENTAL MATERIAL

Data S1.

Supplemental Methods

Bayesian analysis for the increase in the number of operations for complex CHDs

We performed a Bayesian analysis to examine the increase in the number of operations for complex CHDs between the pre-disaster period (2007–2010) and the post-disaster period (2011–2014).

Model specification: For a disease in a year, the number of operations is denoted as *Y* and the number of live births is denoted as *n*. Therefore, the operation rate per year is $\frac{Y}{n}$. Assuming that *Y* follows a Poisson distribution with an expectation *μ* and a linear predictor *z*, which is associated with the operation rate using 'log' as a link function, we obtain

$$\ln\left(\frac{\mu}{n}\right) = z \Leftrightarrow \ln(\mu) = z + \ln(n)$$

where ln(*n*) is an offset term. The pre-disaster operation rate and the rate of change between the pre- and post-disaster periods should differ among CHDs; the linear predictor *z* is then modeled as

$$z = \alpha + \beta \cdot x,$$

where *α* is a parameter of the pre-disaster operation rate, *β* is a parameter of the change rate between the pre- and post-disaster periods, *x* is an indicator variable (pre-disaster: *x* = 0; post-disaster: *x* = 1). *α* and *β* are applicable for each disease, and *x* is applicable for each year. Assuming *α* and *β* are random effects that are normally distributed with their mean values, μ_α and μ_β, respectively, our model is expressed as follows:

$$Y_{ij} \sim \text{NegativeBinomial}\left(p_{ij}, r\right),\ p_{ij} = \frac{r}{\mu_{ij} + r}$$

$$\ln\left(\mu_{ij}\right) = \alpha_i + \beta_i \cdot x_j + \ln\left(n_j\right)$$

$$\alpha_i \sim \text{dnorm}(\mu_\alpha, \tau_\alpha),\ \mu_\alpha \sim \text{dnorm}(0, 10^{-4}),\ \tau_\alpha = 1/s_\alpha{}^2,\ s_\alpha \sim \text{dunif}(0, 10^4)$$
$$\beta_i \sim \text{dnorm}(\mu_\beta, \tau_\beta),\ \mu_\beta \sim \text{dnorm}(0, 10^{-4}),\ \tau_\beta = 1/s_\beta{}^2,\ s_\beta \sim \text{dunif}(0, 10^4)$$
$$r \sim \text{dunif}(0, 10^6)$$

where Y_{ij} the number of operations of disease i in year j, μ_{ij} is an expectation of the number of operations of disease i in year j, α_i is the intercept of disease i, β_i is a parameter of the change rate of disease i between pre- and post-disaster periods, x_j is an indicator variable in year j ($x_1 \ldots x_4 = 0$, $x_5 \ldots x_8 = 1$); n_j is the number of live births in year j. 'dnorm(μ, τ)' indicates a normal distribution with a mean μ and precision τ, and 'dunif(a, b)' indicates a uniform distribution ranging from a to b.

MCMC sampling: We performed MCMC sampling using JAGS 4.2.0 on the R 3.2.3 platform. Four chains were executed in parallel, and each chain included 500000 burn-ins and 500000 trials with a sampling frequency of every 100 trials. We checked the convergence using the multivariate version of $\hat{R}$ [1], which is the potential reduction scale factor. Using the obtained 20000 posterior samples, mean values and quantiles (2.5% and 97.5%) were calculated. Note that the percent changes between the pre- and post-disaster periods were calculated as $(\exp(\mu_\beta) - 1) \times 100$ (%) for the total surgery rate or $(\exp(\beta_i) - 1) \times 100$ (%) for the operation rate of each disease.

Table S1. Number of operations for congenital heart disease in Japan.

No.	Disease	Class	Type	2007	2008	2009	2010	2011	2012	2013	2014
	Year			2007	2008	2009	2010	2011	2012	2013	2014
	Number of births			1,089,818	1,091,156	1,070,035	1,071,304	1,050,806	1,037,231	1,029,816	1,003,539
	Total			4,351	4,505	4,452	4,778	4,944	4,790	4,823	4,815
1	PDA	late	simple	517	595	548	639	502	600	637	663
2	Coarctation (simple)	late	simple	48	37	36	55	62	55	44	69
3	Coarctation + VSD	early	complex	135	118	144	171	154	130	179	145
4	Coarctation + DORV	early	complex	33	26	38	31	25	12	25	38
5	Coarctation + AVSD	early	complex	11	20	18	19	17	14	12	7
6	Coarctation + TGA	early	complex	10	16	12	8	8	13	15	18
7	Coarctation + SV	early	complex	23	32	23	22	44	23	29	32
8	Coarctation + Others	other	other	21	18	23	24	24	13	18	21
9	Interrupt. of Ao (simple)	early	complex	2	6	8	3	5	4	2	8
10	Interrupt. of Ao + VSD	early	complex	64	67	64	88	66	67	81	89
11	Interrupt. of Ao + DORV	early	complex	8	10	21	13	12	11	14	8
12	Interrupt. of Ao + Truncus	early	complex	6	8	2	5	4	7	2	1
13	Interrupt. of Ao + TGA	early	complex	2	6	3	0	3	6	2	2
14	Interrupt. of Ao + Others	early	complex	27	19	20	13	17	21	20	10
15	Vascular ring	late	simple	13	11	13	14	15	17	23	21

16	PS	late	simple	12	14	16	18	18	17	12	12
17	PAIVS or critical PS	late	simple	102	112	125	102	126	118	136	107
18	TAPVR	early	complex	159	208	176	180	186	178	167	189
19	PAPVR ± ASD	late	simple	21	14	15	10	10	4	21	5
20	ASD	late	simple	57	69	67	72	108	65	100	87
21	Cor triatriatum	early	complex	15	17	11	6	10	14	12	16
22	AVSD (partial)	late	simple	16	24	16	23	10	26	20	12
23	AVSD (complete)	early	complex	182	178	189	197	213	212	197	217
24	AVSD + TOF or DORV	early	complex	21	14	17	22	18	22	30	21
25	AVSD + Others	early	complex	9	12	18	10	23	18	13	15
26	VSD (subarterial)	late	simple	134	127	102	125	99	144	139	113
27	VSD (perimemb/muscular)	late	simple	891	874	938	1008	1028	935	974	907
28	VSD + PS	late	simple	24	15	26	47	15	40	17	16
29	DCRV ± VSD	early	complex	13	14	14	22	19	18	16	19
30	Aneurysm of sinus valsalva	other	other	7	4	13	3	10	7	2	1
31	TOF	early	complex	273	273	247	285	335	307	365	301
32	PA + VSD	early	complex	131	136	130	114	161	191	151	145
33	DORV	early	complex	210	188	183	194	196	190	200	214
34	TGA (simple)	early	complex	113	126	146	122	127	121	108	127
35	TGA + VSD	early	complex	46	55	69	63	58	71	55	54
36	TGA VSD + PS	early	complex	29	22	25	18	31	26	26	38

37	Corrected TGA	early	complex	48	20	30	37	23	39	34	60
38	Truncus arteriosus	early	complex	39	41	39	47	47	51	57	53
39	SV	early	complex	340	325	307	364	428	375	352	355
40	TA	early	complex	80	98	72	87	93	70	76	87
41	HLHS	early	complex	217	221	240	238	272	275	234	275
42	Aortic valve lesion	other	other	29	27	44	37	29	29	28	23
43	Mitral valve lesion	other	other	64	49	42	38	36	58	43	32
44	Ebstein	early	complex	38	24	37	35	41	37	41	36
45	Coronary disease	other	other	22	11	11	10	14	12	25	12
46	Others	other	other	89	204	114	139	202	127	69	134

The number of operations for congenital heart disease (under the age of one, excluding 'redo') is shown. The number of births is excerpted from the Population Survey Report published by the Ministry of Health, Labour and Welfare. Disease names and their Nos. are based on the annual reports published by The Japanese Association for Thoracic Surgery.

Figure S1. Result of the Bayesian analysis for complex CHDs.

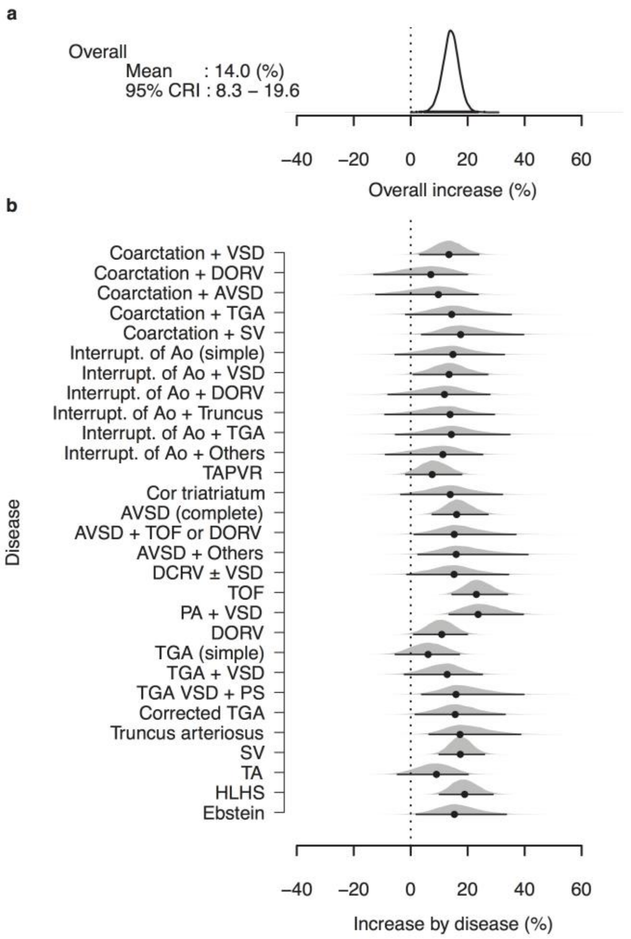

The increase in the number of operations for complex CHDs between 2007—2010 and 2011—2014 is shown. a. Overall increase. b. Each category. The filled circles and solid lines indicate posterior means and 95% CIs, respectively.

Supplemental Reference:

1. Brooks SP, Gelman A. General methods for monitoring convergence of iterative simulations. *J Comput Graph Stat* [Internet]. 2013;7:434–455. Available from: http://www.tandfonline.com/doi/abs/10.1080/10618600.1998.10474787.
